# Application of Hard and Soft Tissue Regenerative Approach in Predictable Management of Peri-Implantitis: A Five-Year Follow-Up Case Report

**DOI:** 10.7759/cureus.60307

**Published:** 2024-05-14

**Authors:** Shaleen Khetarpal, Jaya Nathani, Madhu S Ratre, Mishthu Solanki

**Affiliations:** 1 Periodontology, Government College of Dentistry, Indore, IND; 2 Periodontology, Bhabha College of Dental Sciences, Bhopal, IND; 3 Pediatric and Preventive Dentistry, Smile Makers Dental Clinic, Indore, IND

**Keywords:** implant complications, soft tissue augmentation, guided bone regeneration, peri-implantitis, dental implant

## Abstract

As implant dentistry expands, the number of implants being placed increases, and so does the prevalence of associated complications, resulting in implant failure if not timely attended. The present case report aims to discuss the successful regenerative management of peri-implantitis by both hard and soft tissue augmentation with a five-year follow-up. A 60-year-old male reported a chief complaint of purulent discharge, 7 mm peri-implant probing depth, and radiographic bone loss with no pathologic mobility of the dental implant. The reflection of the full-thickness flap revealed a circumferential defect. Guided bone regeneration (GBR) was performed using a combination of autogenous and alloplastic bone grafts around the implant site. To maintain the peri-implant marginal bone level, soft tissue augmentation was done using the vestibular incision subperiosteal tunnel access (VISTA) approach, after six months. A five-year follow-up showed a significant bone fill and stable soft tissue around the implant clinically and radiographically.

## Introduction

Dental implants have evolved as a reliable treatment option in the management of partial or complete edentulous sites. Osseointegration is the most important predictor in the long-term surgical success of dental implants along with stable implant abutment-soft tissue interface and esthetics outcome. Peri-implant diseases are inflammatory lesions of the surrounding peri-implant tissues and include two different entities, peri-implant mucositis and peri-implantitis [1]. The mean implant-based and subject-based peri-implant mucositis prevalence was 29.48% and 46.83%, respectively, and the mean implant-based and subject-based peri-implantitis prevalence was 9.25% and 19.83%, respectively [2].

The risk factors of peri-implantitis include poor plaque control, smoking, history of periodontitis, adverse occlusal loading, uncontrolled systemic diseases affecting bone, and poor-quality soft tissue [3]. Recently, the effect of the amount and quality of soft tissue around the implant has been considered a very important factor for success.

At present, the suggested treatment plan for peri-implant intra-bony defect includes the establishment of effective plaque control, decontamination of the implant surface, and surgical augmentative procedures for bone regeneration. Definite management of peri-implantitis is still a challenge for clinicians due to the unpredictable outcomes. The present case report discusses the successful regenerative management of peri-implantitis in two stages using guided bone regeneration (GBR) procedure at the first stage and soft tissue augmentation at the second stage with a five-year follow-up.

## Case presentation

A 60-year-old male reported to the Department of Periodontology with a chief complaint of purulent discharge in the left lower back tooth region for two months. He had a history of implant placement with a screw-retained PFM prosthesis in relation to the mandibular left first molar site three years back. The patient was systemically healthy and non-smoker.

**Figure 1:**
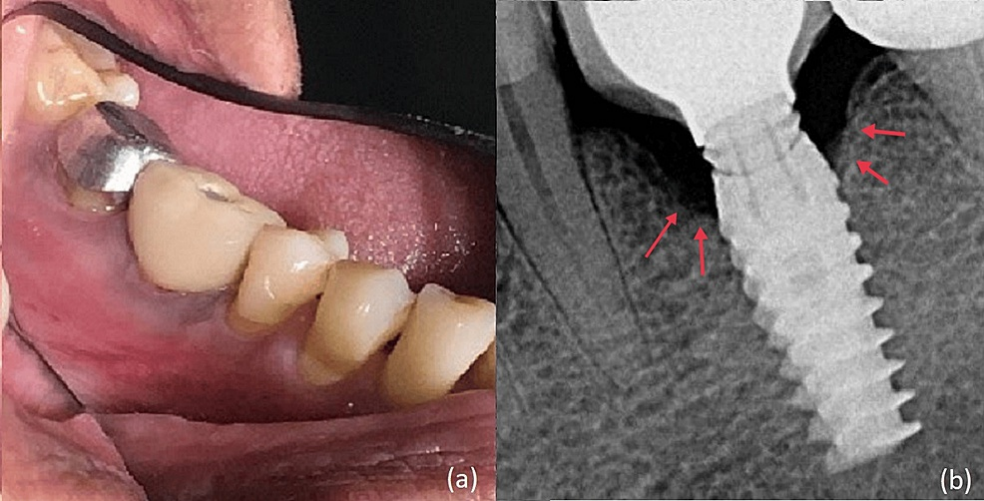
(a) Pre-operative photo of left mandibular first molar showing inflamed peri-implant tissue. (b) Pre-operative radiograph shows a saucer-shaped defect around the implant.

On clinical examination of the implant with respect to 36, bleeding on probing, suppuration, and peri-implant pocket depth of 7 mm was observed. Peri-implant keratinized mucosal width was 1 mm (Figure 1a). Pathological mobility of the implant was absent. The intraoral periapical radiograph revealed a saucer-shaped defect around the implant (Figure 1b). A diagnosis of moderate peri-implantitis was made [4]. Peri-implant tissue was debrided using plastic scalers with copious saline irrigation. Antibiotics Amoxicillin 500 mg and Metronidazole 400 mg eight hourly were prescribed for seven days.

**Figure 2:**
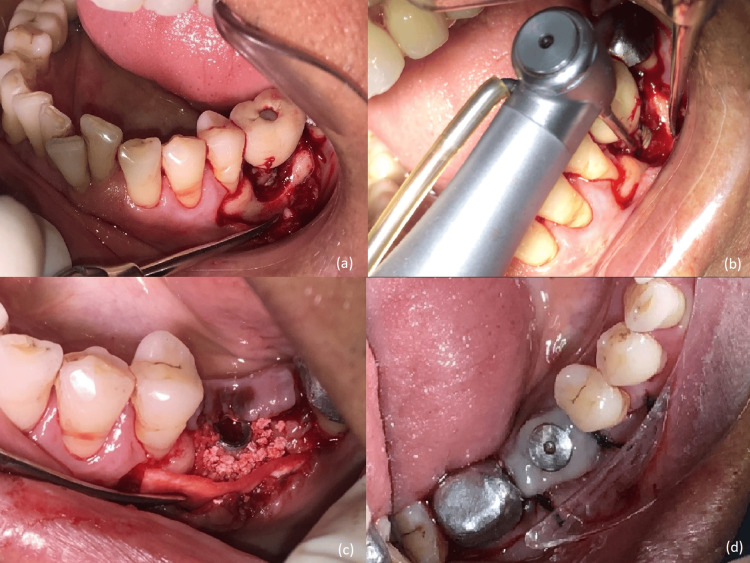
(a) On full-thickness mucoperiosteal flap reflection, a circumferential defect around the implant is seen. (b) Decontamination of implant surface done using titanium brush and copious irrigation. (c) Placement of autogenous bone graft layered by alloplastic bone graft covered with a resorbable collagen membrane. (d) Customized gingival former placed.

**Figure 3:**
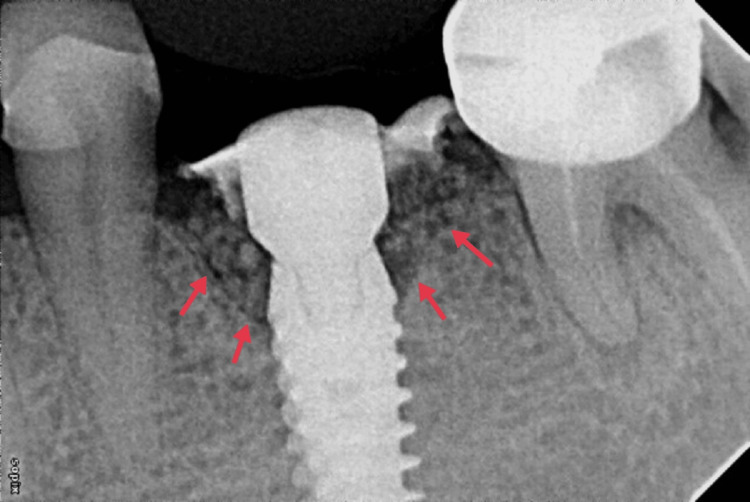
Immediate post-operative radiograph.

On clinical evaluation on the third day, there was no active purulent discharge present. GBR was planned for the management of peri-implant bone loss. Written consent and necessary blood investigations were obtained. A full-thickness flap was reflected; after profound anesthesia and debridement, a circumferential saucer-shaped osseous defect around the implant was visible (Figure 2a). The implant surface was thoroughly decontaminated with a Teflon-coated titanium brush and copious irrigation with saline (Figure 2b). GBR was performed using a combination of autogenous and synthetic bone grafts with a resorbable membrane. An autogenous bone graft was obtained from the ramus of the same side by scraping method. It was placed over the implant followed by a layer of alloplastic bone graft HA-βTCP (Osteon II, Dentium, USA). The graft was protected with a resorbable cross-linked collagen membrane (Collagen Membrane, Dentium, USA) (Figure 2c). Customized healing abutment (using flowable composite around conventional healing abutment) was placed over the implant at the grafted site (Figures 2d, 3). The site was sutured using simple interrupted sutures and analgesics, antibiotics were continued, and oral hygiene instructions were reinforced. Sutures were removed after two weeks. Postsurgical healing was uneventful. The patient was recalled at regular intervals of three months.

**Figure 4:**
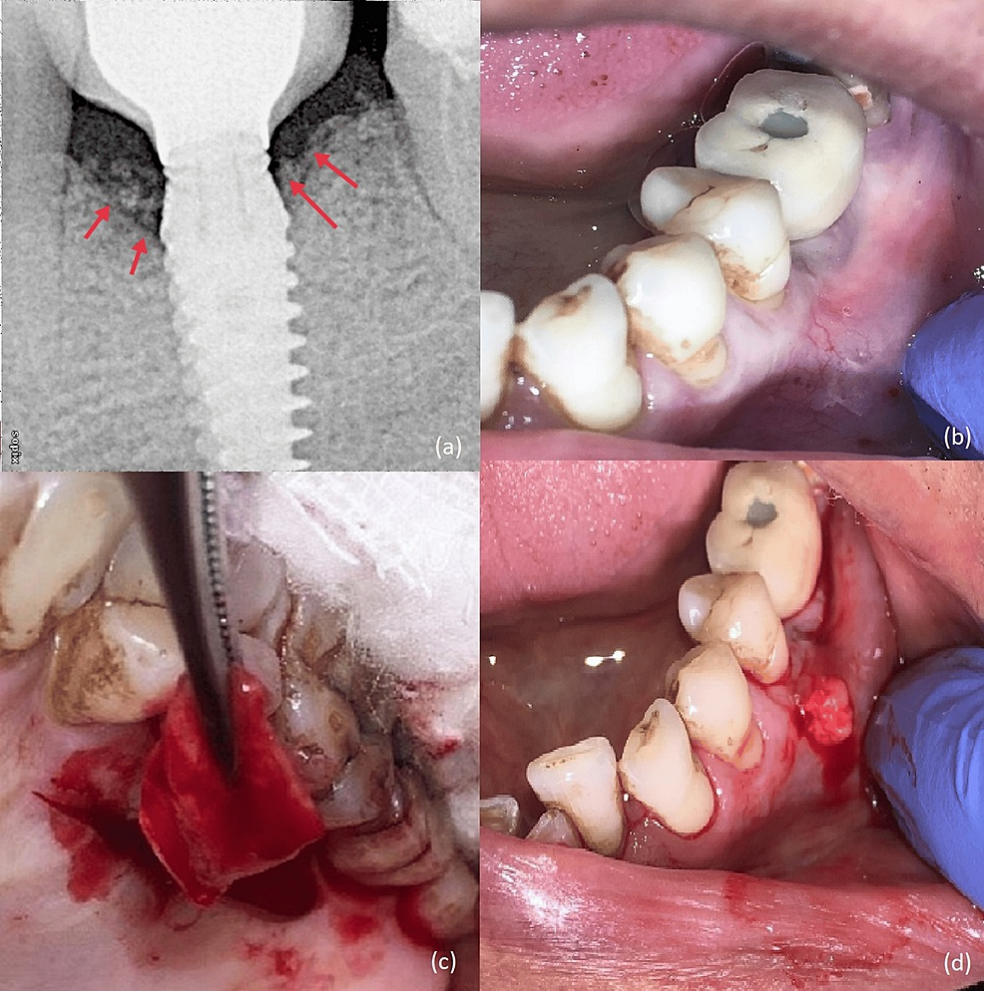
(a) Six-month post-operative radiograph. (b) A greyish hue of buccal mucosa was observed. (c) Harvesting autogenous SECTG from palate. (d) Placement of the autogenous connective tissue graft (VISTA technique). VISTA, vestibular incision subperiosteal tunnel access; SECTG, subepithelial connective tissue graft

At the sixth-month visit, approximately 2 mm of bone fill was evident on radiographic evaluation (Figure 4a). Implant loading was done. At this stage, a greyish hue of the buccal mucosa was observed suggestive of thin peri-implant soft tissue (Figure 4b). To maintain the stability of the peri-implant marginal bone level and improve the quality of peri-implant mucosa, soft tissue augmentation around the implant was planned. Vestibular incision subperiosteal tunnel access (VISTA) along with subepithelial connective tissue graft (SECTG) from the palate was done (Figures 4c, 4d).

**Figure 5:**
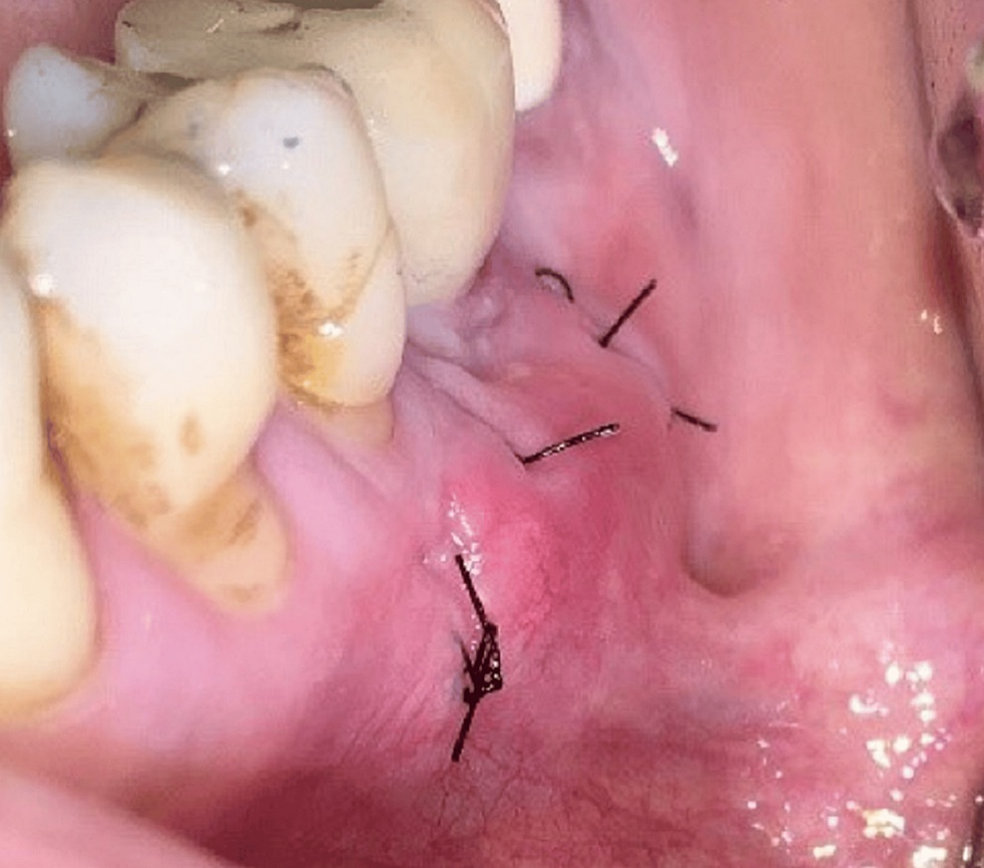
Two weeks after soft tissue augmentation surgery.

**Figure 6:**
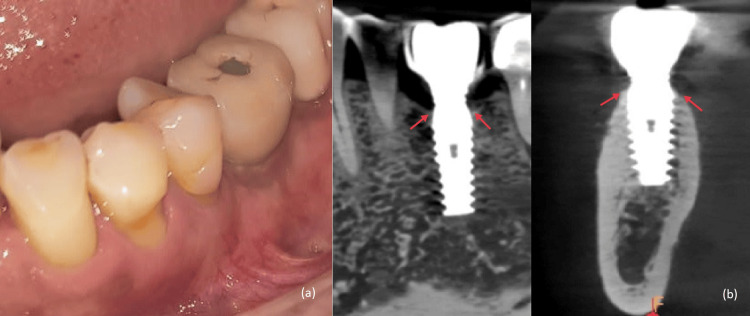
(a) Two-year post-operative clinical photograph. (b) Two-year post-operative CBCT scan. CBCT, cone-beam computed tomography

**Figure 7:**
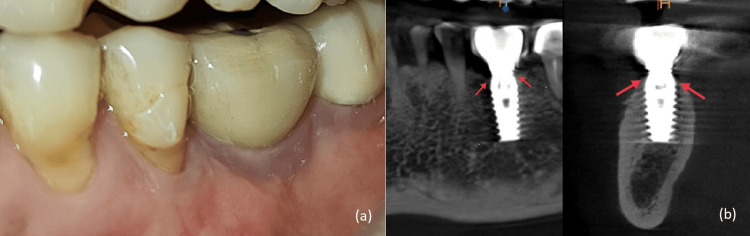
(a) Five-year post-operative clinical photograph. (b) Five-year post-operative CBCT scan. CBCT, cone-beam computed tomography

Sutures were removed on the 14th day (Figure 5), and healing was evaluated on a regular basis at three-month intervals for the first year and yearly basis after that (Figures 6a, 6b). The volume and thickness of buccal mucosa had increased and the soft tissue color and consistency were improved from greyish hue to pink. During the five-year follow-up on clinical and radiographic evaluation, the peri-implant soft tissue and bone levels were found to be stable and satisfactory (Figures 7a, 7b).

## Discussion

Peri-implant mucositis and peri-implantitis are becoming the most prevalent diseases in dentistry. In a recent study evaluation of 6129 implants done in 2127 patients, 1/5th of all implants and 1/3rd of the patients were observed with peri-implantitis [5]. Treatment of peri-implantitis without delay is of paramount importance because the rate of peri-implant tissue destruction is faster, as the arrangement of collagen fibers around the implant is parallel with lower vascularity in comparison to natural teeth. Studies have shown a non-linear and accelerating progression of peri-implantitis where the rate of destruction increases with time [6].

Decontamination of the implant during surgery is an imperative step for the removal of the plaque layer. In the current case, titanium brushes were used because of their close adaptability to the microstructure of the implant surface [7]. 

Augmentative surgery was executed for peri-implant intra-bony defect. GBR for a peri-implant defect involving less than half of the implant fixture and >2 mm bone defect around the implant has been recommended in the literature [8,9]. Albeit being the gold standard due to its osteogenic, osteoconductive, and osteoinductive properties, due to limited availability and faster resorption of autogenous bone graft, alloplast was also used in the present case. Layering of autogenous bone graft toward implant surface and alloplast over it was done [10]. This facilitates autogenous bone contact with the implant surface to promote regeneration of bone. The placement of a resorbable collagen barrier membrane provides space maintenance for the improved outcome. In a clinical trial conducted by Wiltfang et al., it was established that bone grafting in peri-implant defects results in predictable results [11]. 

A recent consensus has concluded that concomitant soft and hard tissue augmentation procedures significantly reduce marginal soft tissue recession compared to hard tissue augmentation alone [12]. Application of VISTA with SECTG being a minimally invasive surgical approach enabled to avoid exposure of recently bone grafted site. It facilitated the achievement of better graft survival and color match and significantly improved the thickness of soft tissue around the implant along with preventing marginal tissue recession [13]. Puisys & Linkevicius both demonstrated statistically significant less marginal bone loss with thick tissue or augmented thin tissues [14]. A systematic review reported that the performance of soft tissue grafting procedures for gain of mucosal thickness resulted in significantly less interproximal marginal bone loss over time [15]. The stable soft and hard tissue status could be appreciated clinically and radiographically (CBCT) during the five-year-follow up.

## Conclusions

GBR procedure followed by soft tissue augmentation to increase the horizontal thickness of soft tissue is a successful method of management of peri-implantitis. Also, the thickness of soft tissue around the implant is an important parameter for long-term maintenance of implants, and further research should be done to explore this area. Challenges in the treatment modality include it being technique-sensitive and involving the use of both hard and soft tissue procedures; hence, the operator’s knowledge and experience play a key role. Also, timely diagnosis and treatment are necessary as delay leads to progressive bone loss and implant failure. 
